# Screening for depression among a sample of US college students who engage in recreational prescription opioid misuse

**DOI:** 10.15171/hpp.2020.10

**Published:** 2020-01-28

**Authors:** Robert E. Davis, Martha A. Bass, M. Allison Wade, Vinayak K. Nahar

**Affiliations:** ^1^Substance Use and Mental Health Laboratory, Department of Health, Human Performance and Recreation, University of Arkansas, 155 N. Stadium Dr. HPER 310B, Fayetteville, AR 72701, USA; ^2^Department of Health, Exercise Science Recreation Management, University of Mississippi, 218 Turner Center, University, MS 38677, USA; ^3^Department of Preventive Medicine, University of Mississippi Medical Center, Jackson, MS, USA; ^4^Department of Dermatology, University of Mississippi Medical Center, Jackson, MS, USA

**Keywords:** Depression, Marijuana, Opioid, Sadness, Students

## Abstract

**Background:** Among student populations, literature has identified associations between prescription opioid misuse and symptoms of depression such as hopelessness, sadness, and emotional pain. Thus far, existing literature has yet to investigate associations between prescription opioid misuse and depression using validated screening instruments for depression when exploring such associations. The purpose of this study was to utilize a validated screening tool to explore quantifiable presence of depression among college students who engage in recreational prescription opioid misuse (RPOM). Additionally, gender differences in depression and co-occurring substance use are examined.

**Methods:** Students (n = 104) of a large university in the Southeastern United States who reported ROM within the past 6 months completed instrumentation assessing demographics, substance use, as well as, screening tools for depression and possible opioid use disorder (OUD).

**Results:** Positive depression screens were significantly higher among females, however, nearly56% of participants screened positive for major depression. Though high levels of co-occurring substance use were observed among the entire sample, males were significantly more likely to report cocaine use, more frequent use of alcohol and marijuana, as well as, exhibit a positive screen for disordered opioid use, at a rate 5 times that of their female counterparts.

**Conclusion:** Students who engage in RPOM are a particularly heightened-risk subsample of the college population who exhibit high levels of depressive symptomatology and substance use behavior. Targeted programming and further investigations are needed among this specific population. Future studies are encouraged to utilize validated instruments when assessing depression among students.

## Introduction

Nationally representative data from the United States suggests that significantly higher percentages of individuals 18-25 years misuse prescription medications compared to younger or older age groups.^1^ In 2015, nearly 1 million individuals received treatment for prescription opioids, 167 000 being 18-25 years.^1^ Prevalence rates for lifetime misuse of prescription opioids among college samples vary greatly by study,^2,3^ with estimates as high as 32%.^4^ More proximal recall periods produce estimates, typically, much lower at less than 10%.^5,6^ Pressure from the academic environment, perceived peer approval and behavior, also the large and diverse social networks of college students may contribute to opioid misuse.^1,7-10^ Although students have reported misusing opioids for the treatment of emotional pain, it appears that motivation for misuse is largely recreational.^2,6,11^

Characteristics specific to college students place them not only at risk for substance use but also to mental health complications, particularly depressive symptoms.^12^ Many students are experiencing their first glimpse of independence, which comes with the loss of their family unit, proximal support system, and may lead to risky decision making.^13^ Additionally, school related stress has been shown to associate with depression among this population.^14^ The patient health questionnaire 9 (PHQ-9) is a common screening tool for major depressive disorder (MDD) which has been validated against physician diagnosis and is used in research and clinical settings.^15,16^ Among a general sample of approximately 14 000 college students recruited from universities across the United States, 9% exhibited positive screens for MDD utilizing the PHQ-9 instrument.^12^ Review findings from studies incorporating a multitude of depression scales provide high prevalence estimates of depression of any form among college students, weighted mean rate of 30.6%, and further suggest that rates among this population may be higher than those among the general population.^17^ Further, substance use has also been shown to deteriorate the psychological health of students.^12,13^ Particularly, prescription opioid misuse has been linked to suicidality^1,18^ and has been associated with the initiation of depression,^19^ making recreational opioid misuse a concerning form of substance use behavior among students.

Recent attention given to the current opioid crisis has spawned literature indicating a relationship between opioid misuse and the existence of depressive symptoms and other affective dysregulation among college students.^6,20,21^ Currently, these studies have assessed only particular depressive symptomatology, such as, hopelessness, sadness, emotional pain, and suicidal ideation. As the literature, thus far, has excluded use of validated depression screening instruments, it is yet unknown to what extent depression may actually exists among students who misuse prescription opioids.

Therefore, the current study was exploratory and held three aims. First, to fill a gap in the literature by assessing a presence of depression among college students who engage in recreational prescription opioid misuse (RPOM) while utilizing a validated screening tool for MDD. Gender-based differences have been presented in the general literature regarding the existence of depression^17,22,23^ and misuse of abusable prescription drugs.^24^ Thus, a second study aim was to examine such gender-based differences in depression and substance use among this sample of opioid misusers. Third, to explore the presence of disordered opioid use among this sample, as opioid use disorder (OUD) may exacerbate existing mental health problems.^25^

## Materials and Methods

### Participants and procedures 

Data for the current study were collected in the fall of 2017 and in conjunction with a larger study investigating the belief system underlying RPOM.^26^ Ethical approval for all procedures was obtained from the institution’s ethics committee prior to participant contact. Participants were students of a large public university located within the Southern United States. Individuals reporting past six month RPOM were identified from the larger sample and asked additional questions specific to the aims of this study (i.e. depression and use substance items). These individuals (n = 104) reporting past 6-month RPOM comprised the current study sample. For recruitment, the University’s institutional research office provided 5000 random email addresses with equal representation by gender and academic classification. Recruitment emails provided informed consent and an invitation to participate. Students were informed that by completing the survey they would be eligible to enter a drawing for one of several gift cards. Those agreeing to participate opened a link taking them to the electronic survey where they provided responses to questionnaire items. Response rate from the initial 5000 recruitment emails was approximately 20%. Following removal of those with large amounts of missing data, roughly 10% (104) of the successfully recruited students reported past 6-month RPOM, thus meeting the inclusionary criteria for the current study.

Prior to answering specific questions, participants were introduced to an operational definition of “recreational misuse” (i.e. to get high, for euphoric effects, to have fun, relax, or experiment). Students were also provided examples of drugs qualifying as opioid pain relievers (i.e. opioids like Vicodin, OxyContin, Tylenol 3, Percocet, Darvocet, buprenorphine, morphine, hydrocodone, oxycodone, methadone, fentanyl, or other such opioids). Questionnaire items assessed factors such as demographics, misuse of opioid medications and other substances, as well as, screener instruments for depression and possible OUD. Upon completion of the survey, participants were presented with a link to the University’s counseling support center where they could find information about available resources should they, or someone they know, be dealing with a mental health or substance use problem.

### Measures

*Substance use*

RPOM was assessed by one item identifying a temporal trend pertaining to misuse behavior. The item asked “How frequently have you used prescription opioid medications (i.e., opioids like Vicodin, OxyContin, Tylenol 3, Percocet, Darvocet, buprenorphine, morphine, hydrocodone, oxycodone, methadone, fentanyl, or other such opioids) for recreational purposes in the past six months?” Participants responded using 7-point scale (1-7) ranging from “1” never, “2” once, “3” more than once, “4” every few months, “5” every month, “6” every week to “7” most days. Use of other common substances (i.e. alcohol, marijuana, methamphetamine, heroin, ecstasy, and non-opioid prescription drugs) over the past six months were also assessed. Each substance was evaluated on the previously mentioned 1-7 scale, responses ranging from “1” never to “7” most days.

*Depressive symptomatology*

The Patient Health Questionnaire (PHQ-9) was used to assess depressive symptomatology. The PHQ-9 is a screening and diagnostic instrument for MDD, widely utilized in both clinical and research settings.^27,28^ The PHQ-9 consists of 9 items each addressing specific symptoms of depression. The patient indicates whether they have been subject to any of the stated symptoms of depression over a period of the past two weeks. Sample items include “little interest or pleasure in doing things”, “feeling bad about yourself, that you are a failure, or have let yourself or your family down”, and “thoughts that you would be better off dead or hurting yourself in some way”. The participant assigns a score of 0 to 3 indicating how often they have been bothered with the symptom, with “0” being not at all to “3” nearly every day. Possible scoring ranges from 0 to 27. The cut point of ≥10 was chosen to indicate a positive screen for MDD as this threshold has demonstrated validity against clinical diagnosis of MDD by mental health practitioner.^16,27^ As scores increase from 10 to 27, MDD moves from mild to severe. It is important to note, we are not diagnosing individuals, only identifying positive and negative screens. In the current study, Cronbach’s alpha for the PHQ-9 subscale was 0.85.

*Opioid use disorder*

OUD is identified by evidence of impaired control, social dysfunction, risky use, as well as physiological and affective problems associated with the use of opioid drugs. The 11 diagnostic criteria for OUD outlined by the Diagnostic and Statistical Manual for Mental Disorders, 5^th^ edition (DSM-V) were directly used to create an 11 item subscale. Participants responded “yes” or “no” to each criterion’s presence over the recall period. For example, “Important social, occupational or recreational activities are given up or reduced because of my opioid use” and “I have a persistent desire or unsuccessful efforts to cut down or control my opioid use”. According to the DSM-V, manifestation of 2 or more of the criteria indicates the possible presence of OUD. As we were not diagnosing individuals, answering yes to 2 or more items was treated as a positive screen for OUD of at least a mild state. In the current study, Cronbach’s alpha for this subscale was 0.80.

### Data analysis

Descriptive statistics were computed and presented for study variables. Further, statistically significant gender-based differences in positive screens for disordered use and depression, as well as, co-occurring substance use were investigated. RPOM, alcohol, marijuana, and non-opioid prescription drug misuse were evaluated by frequency. Due to restricted variance across frequency scales, cocaine and ecstasy were evaluated on the basis of any use during the recall period. Similarly, few reports of heroin and methamphetamine use were observed, as such, gender differences were not tested herein. Differences among ordinal variables were evaluated using Mann-Whitney U-test, with differences in binary variables calculated using Pearson’s chi-square test of independence. Alpha levels for significance tests were set at 0.05 and all analytic procedures were carried out using IBM SPSS Statistics, version 24.0. IBM Corp. Armonk, NY.

## Results

### Demographics

Participants of the current study were university students (n = 104), of both undergraduate and graduate status, who self-reported RPOM within the last six months. As seen in Table 1, the mean age of participants was 22.1 years (SD = 5.9). This sample exhibited a fairly even distribution by gender with 57 males and 47 females. Because of limited representation by particular ethnic groups, race/ethnicity was collapsed to represent whites and members of a minority group. The participants identified themselves as predominantly white at 87 (83.7%), while 17 participants identified as a racial minority. The largest segment of students per academic classification were juniors at the university (28.8%). Further, less than half of participants reported a grade point average less than 3.0.

### Depression

Approximately, 56% of our sample met or exceeded the initial cut point for MDD (i.e. ≥10), as designated by the PHQ-9 diagnostic instrument (Table 2). Though not shown in tabular form, a score ≥15 was observed for 26.9% of participants indicating moderate to severe MDD. As seen in Table 2, the prevalence of MDD was significantly higher among females compared to males at 68.1% and 45.6%, respectively (*P* = 0.022).

### Substance use behaviors

As inclusionary criteria, all participants reported RPOM at least once in the past six months. As seen in Table 2, no significant difference was observed between males and females in regard to RPOM frequency (*P* = 0.793). Among our sample, use of alcohol, marijuana, and non-opioid prescription drugs were common, at 89.4%, 73.1%, and 52.9%, respectively, Table 2. Fewer individuals reported use of the drugs cocaine (38.5%), ecstasy (21.2%), and methamphetamine (7.7%). Encouragingly, only one individual reported past six months use of heroin. Though no gender difference was identified for frequency of RPOM or use of non-opioid prescription drugs, significant differences were observed for other associated drug use behaviors, Table 2. Specifically, males reported more frequent alcohol (*P* = 0.012) and marijuana (*P* = 0.001) use and were more likely to report cocaine use (*P* = 0.040) than their female counterparts. As noted, methamphetamine and heroin use were not evaluated on the basis of significant gender differences. However, it is worthy of note that only one female reported methamphetamine use and no female reported heroin use during the study period.

### Opioid use disorder

Seventeen participants (16.3%) screened positive for OUD of at least mild severity, Table 2. Our analysis identified an association between gender and screening positive for OUD (*P* = 0.016). Specifically, the odds of a positive OUD screen were nearly 5 times higher for males when compared to female misusers. Of interest, the gender difference in OUD exists in absence of a statistical difference in RPOM frequency between males and females.

## Discussion

This study identifies staggering mental health problems among a specific high-risk population, college students who engage in RPOM. The high levels of depression were identified by a screening instrument exhibiting robust validity against clinical diagnosis.^16^ Specifically, 55.8% of the over-all sample screened positive for MDD of at least mild severity. Positive MDD screens were more common among female participants. Additionally, 16.3% of participants in the current study met classification criteria for OUD of at least mild severity, the majority of which were males. Collectively, these findings indicate mental health complications as an important correlate of RPOM among college students.

A main strength of the current study was its use of validated instrumentation for the assessment of depression. Few studies have produced correlations between opioid misuse and depressive symptomology,^6,11,18,21,29^ however, to our knowledge no studies have attempted to identify a prevalence of depression among college students who misuse opioids through an established screening instrument. Specifically, coping with depression, the treatment of emotional pain, and treatment of psychiatric distress have been identified as motives for opioid misuse among college students.^6,11,29^ Additionally, prescription opioid misuse has been associated with increased odds of experiencing depressive symptoms such as hopelessness, sadness, and suicidality among this population.^18,21^ It is important, not only for validity, but also for comparative purposes that studies attempt to utilize validated instrumentation when measuring depression among their samples.

In the current study, estimates of MDD greatly exceeded estimates among college students previously reported by studies utilizing the same instrumentation.^12,22,30^ Among a sample of general college students, positive MDD screens for 5.2% and 4.1% of undergraduate and graduate students, respectively, were reported.^22^ Another study of over 14 000 students from 26 universities across the United States produced positive MDD screens for 9% of participants.^12^ Additionally, a study of medical students reported 14.3% scoring 10 or above on the PHQ-9.^30^ For perspective, a recent study reported that among chronic non-cancer pain patients (n = 785) undergoing chronic opioid therapy, 14.6% screened positive for MDD utilizing the PHQ-9 and cut points applied by the current study.^28^ Consistent with our findings, literature shows females as more prone to MDD.^12,17,22,23,30^ A systematic review of depressive prevalence among university students identified only one study where males exhibited greater risk.^17^ Prospective study among adolescents suggests that young females are more reactive to stress and thus more prone to development of depression.^31^ As college is a high stress environment and the fact that evidence suggests a bidirectional relationship between depression and opioid use,^32^ this may partially explain our findings.

To the best of our knowledge this was also the first study to examine the presence of OUD explicitly among a college sample. We recommend that future study incorporate measures of disordered use when studying this behavior to better understand the deleterious effects these drugs impose on student’s psychosocial functioning. OUD is a common correlate of long-term drug therapy.^25^ Our OUD findings, at 16%, are concerning as these are not daily medical users. Previous study indicates that as many as half of individuals with OUD have co-occurring MDD.^33^ Similarly, in our sample, just more than half of those screening positive for OUD also exhibited positive screens for MDD. In the current study, significantly more students screening positive for OUD were male which may reflect DSM-V reports that males with mental health disorders are more likely to have a co-occurring substance use disorder.

RPOM frequency was generally low among the entire sample, however, attempting to quantify frequency was of interest being that studies among this population typically treat opioid use as a dichotomy.^5,6,11,34^ Interestingly, we observed no significant difference in RPOM frequency between males and females, however, significant differences were identified for alcohol, marijuana, and cocaine use. As opioid effects are intensified when used in combination with other substances,^35^ this finding may not only indicate males at increased risk for RPOM outcomes such as overdose, but may have caused males to better associate RPOM with the physical, psychological, and social impairments addressed by the OUD screening items.

Taken collectively, gender differences in MDD, OUD, co-occurring substance use, and a lack of difference in frequency of RPOM may highlight incompatible determinant factors for recreational misuse. Though speculative, our operational definition of “recreational misuse” may have created differential associations in the minds of participants. For example, “to relax” may have been perceived as a means of maladaptive coping to escape negative psychological states among females (e.g. self-medication of stress or sadness), whereas males may have related “recreational misuse” to other elements presented in the operational definition (i.e. have fun, experience euphoric effects, etc). Furthermore, we did not assess the dosage of opioids consumed which may have partially explained our findings pertaining to OUD. Reliability of this information would be problematic when quantifying morphine equivalents among recreational users. Indeed, if euphoric effects are the goal of RPOM, one may be inclined to consume higher dosage.

There were several limitations to address in the current study. This study included a rather small sample of college students (n = 104) from a single university which may impact the generalizability of findings. However, the randomized recruitment strategy utilized may aid in countering this limitation. It is important to indicate, as outlined in the methodology, the 104 students reporting past-6 month RPOM constitutes approximately 10% of participants successfully recruited through a randomized process. Studies among college students assessing prescription opioid misuse over recall periods of one year or less typically produce estimates of misuse at less than 10%.^5,6^ Thus, deriving a larger sample would have been difficult among this population. Further, the self-report nature of this study may have resulted in recall bias, particularly with regard retrospective frequency. Another limitation regarding frequency was the fact that the scale adapted from^36^ included once choice identified as “more than once”. Though instructed to read each response carefully prior to choosing an option, participants may have seen this choice and selected it without considering the further alternatives. Should this, indeed, be the case RPOM may have occurred more frequently than reported among this sample of students. Moreover, we cannot provide causal linkage between variables as this study was cross-sectional in nature.

## Conclusion

Findings suggest that gender differences exist in both depression levels and co-occurring substance use among college students who engage in RPOM. In general, females were more likely than their counterparts to exhibit a positive screen for MDD. While no differences were identified in RPOM frequency between groups, males tended to engage in more non-opioid substance use. Regardless of significant gender differences, positive screens for MDD as well as substance use behavior were high among males and females highlighting a need for specialized intervention efforts among this high-risk subpopulation of students. Future study should attempt to procure larger samples and longitudinal cohort designs capable of probing temporal relationships between RPOM and mental health dysregulation throughout college progression.

## Ethical approval

All study procedures were reviewed and approved by the Institutional Review Board (IRB) at the University of Mississippi. Participants provided their consent prior to participation.

## Competing interests

The authors declare that they have no competing interests.

## Funding

No external funding sources were used for conducting the current research.

## Authors’ contributions

Conception and design: RED and MAB; instrumentation: RED; data collection: RED and AFW; analysis and interpretation: RED and VKN; All authors have contributed to and approved of this manuscript.

## Acknowledgments

The study authors would like to acknowledge the students who participated in the current study and thank them for their contribution to science.

**Table 1:** Descriptive statistics and major depressive disorder (N=104)

**Variables**		**MDD** ^a^
Age, mean (SD)	22.1 (5.9)	
Gender, No. (%)		
Female	47 (45.2)	32 (68.1)
Male	57 (54.8)	26 (45.6)
Race/ethnicity, No. (%)		
White/Caucasian	87 (83.7)	46 (52.9)
Others	17 (16.3)	12 (70.6)
Greek affiliation, No. (%)		
Fraternity/Sorority	40 (38.5)	25 (62.5)
Non-Greek	64 (61.5)	33 (51.6)
University status, No. (%)		
Freshman	23 (22.1)	14 (60.9)
Sophomore	17 (16.3)	7 (41.1)
Junior	30 (28.8)	17 (56.7)
Senior	18 (17.3)	11 (61.1)
Graduate student	16 (15.4)	9 (56.3)
GPA^b^, No. (%)		
<2.0	2 (1.9)	2 (100)
2.0–2.4	9 (8.7)	5 (55.6)
2.5–2.9	23 (22.1)	16 (69.6)
3.0–3.4	36 (34.6)	19 (52.8)
3.5–4.0	34 (32.7)	16 (47.1)

^a^MDD represents a score equal to or surpassing the PHQ-9 cut point of 10 which indicates positive screen for major depressive disorder. ^b^GPA represents grade point average.

**Table 2:** Gender differences in depression, disordered use, and co-occurring substance use

**Variables**	**Total** **% reporting**	**Female (n=47)**	**Male (n=57)**	**χ** ^ 2 ^	**OR (95% CI)**
		**% reporting**	**% reporting**		
MDD^a^	55.8	68.1	45.6	5.27*	0.39 (0.18–0.88)
OUD^b^	16.3	6.4	24.6	6.23,*	4.78 (1.28–17.80)
Cocaine	38.5	27.7	47.4	4.23*	2.35 (1.03–5.37)
Ecstasy	21.2	12.8	28.1	3.62	2.67 (0.95–7.49)
	**Total** **% reporting**	**Median**	**Median**	**U**	**Effect Size**
Prescription opioid misuse frequency	100	3.0	3.0	1301.50	0.001
Alcohol use frequency	89.4	5.0	6.0	972.00*	0.062
Marijuana use frequency	73.1	3.0	5.0	830.00**	0.112
Non-opioid prescription drugs misuse frequency	52.9	1.0	3.0	1148.50	0.017

Abbreviations: OR, odds ratio; CI confidence interval. *Note.* Effect size measured as Eta^2^. ^a^Positive screen for major depressive disorder (MDD) classified by a PHQ-9, not physician diagnosis. ^b^Positive screen for opioid use disorder (OUD). ^c^Fisher exact test. * *P* < 0.05; ** *P* < 0.01.
